# Can China’s vehicular emissions regulation reduce air pollution?—a quasi-natural experiment based on the latest National Vehicular Emissions Standard (stage-VI)

**DOI:** 10.1007/s11356-023-30105-7

**Published:** 2023-10-13

**Authors:** Xing-yuan Liu, Ling-xia Xu, Xiao-qing Wu, Hong-xing Wen

**Affiliations:** 1 School of Economics and Management, Ningxia University, Yinchuan, 750021 People’s Republic of China; 2 School of Economics, Institute of Guangdong High Quality Development, National Economics Research Center, Guangdong University of Finance & Economics (GDUFE), Guangzhou, 510320 People’s Republic of China; 3 International College, Dhurakij Pundit University, 110/1-4 Prachachuen Rd., Laksi, Bangkok, Thailand; 4 School of Accounting, Guangzhou Colleage of Technology and Business, Guangzhou, 510850 People’s Republic of China

**Keywords:** Vehicular emissions standard, DID, AQI, Air pollution reduction

## Abstract

The existing evidence on the environmental effects of vehicular emissions regulation almost comes from developed countries, but the effectiveness of this policy tool in developing countries, especially in China, remains unclear. This study, for the first time, examined the mitigating effects of China’s vehicular emissions regulation on air pollution at the prefecture level cities, by using the latest implementation of China’s National Vehicular Emissions Standard VI (CHINA-VI) as a quasi-natural experimental process of policy shocks. To this end, monthly data from 2018 to 2020 was applied to construct a difference-in-differences (DID) model. The results showed that pilot cities’ air quality index (AQI) significantly decreased by 4.74 compared to non-pilot cities after the implementation of CHINA-VI. Also, the concentration of PM_2.5_, PM_10_, and O_3_ has decreased by 3.6 μg∕m^3^, 6.4 μg∕m^3^, and 3.0 μg∕m^3^, respectively, which means the new China’s vehicular emissions regulation has comprehensively improved air quality. The findings are still valid after a series of robustness tests using different estimation methods such as PSM-DID and IV-2SLS. In addition, we also found heterogeneity in the environmental performance of CHINA-VI across cities. Specifically, cities with lower levels of green finance development and public environmental concern showed a greater emissions reduction effect, but smart cities showed a greater emissions reduction effect than non-smart cities.

## Introduction

In 2015, the United Nations formulated the Sustainable Development Goals (SDGs), which aims to thoroughly address sustainable development in the three dimensions of society, economy, and environment through global efforts in a coordinated manner by 2030. SDG 3, SDG 7, and SDG13, for instance, are committed to “ensuring a healthy lifestyle and promoting the well-being of people of all ages,” “ensuring access to affordable, reliable, and sustainable modern energy,” and “taking urgent action to address climate change and its impacts,” respectively. Obviously, achieving these SDGs requires to substantively advance the sustainable development agenda at all levels of consumption and production, but this is not an easy task for both developed and developing economies. As the largest developing country, China’s number of civil vehicles has increased 1.46 times in the last decade, reaching 311.9 million by 2022, which has led to increasing criticism of environmental pollution and public health risks derived from increased exhaust emissions. Studies have shown that motor vehicle emissions are responsible for 20–67% of the increase in CO concentrations and 12–36% of the increase in NOx concentrations in China (Wei et al. 2008; Zheng et al. 2009). Compared with other industrial exhaust, the health hazards caused by automobile exhaust to residents are more direct (Guo et al. 2016). Therefore, controlling vehicular emissions could significantly reduce the rate of air pollution-related diseases and mortality (Shindell et al. 2011).

Actually, policy-makers have sought various environmental regulations to balance the conflict between environmental governance and economic development, including control and command (CAC) regulation and market-based instruments (MBIs) (Zhu et al. 2023). Although MBIs leave freedom for emitters to choose the least-cost solutions to control pollutants, the Chinese government insists on a CAC policy framework for controlling vehicular emissions. As early as 2001, the Chinese government implemented the National Vehicular Emissions Standard I (CHINA-I) nationwide, referring to the European-I standard. Recently, with the rapid growth of demand for motor vehicles and the heavy burden of exhaust emissions, the Chinese government has decided to implement the CHINA-VI standard, which is known as the strictest vehicular emissions standard in China. Different from the previous vehicular emissions standards (such as CHINA-V), the CHINA-VI has been divided into two stages, namely, “phase A” and “phase B,” which implemented on July 1, 2020, and July 1, 2023, respectively. However, some qualified cities, such as Beijing, Shanghai, Guangzhou, Shenzhen, and Tianjin, have directly implemented the phase B standard and skipped the phase A standard.

Comparing the latest motor vehicle emission standards in Europe and the USA, the pollutant emission limits of CHINA-VI are stricter than EU-VI, but it is unable to surpass LEV-III in the USA, which is close to zero emission. How effective will this strictest vehicle emissions regulation standard be in improving air quality? Does this effect vary across regions? These questions need to be tested through empirical evidence.

Numerous empirical evidence has shown that the implementation of European and American motor vehicle emissions standards can reduce emissions of pollutants such as nitrogen dioxide and PM_2.5_ (Vijayaraghavan et al. 2012; Von Schneidemesser et al. 2017), but the evidence on the efficacy of such policies in developing economies remains scant. As a developing country that implemented vehicular emissions regulation relatively early, the pollution reduction effect of Chinese vehicle emissions standards has received some scholars’ attention, and most of them support its positive role (Ji et al. 2011; Jin et al. 2012; Lang et al. 2012; Wang et al. 2010). However, these studies focused on early gasoline standards implemented before CHINA-IV, and the evidence provided came from locally developed areas, such as the Beijing-Tianjin-Hebei regions. In theory, stricter vehicular emissions standards could be effective in improving air pollution, but it could also lead to other problems. For example, many people will buy new energy vehicles that are more environmentally friendly, but China currently relies mainly on coal-fired power generation, and a large increase in new energy vehicles will lead to a boost in electricity demand. As a result, although the direct emissions of vehicles are reduced, the emissions transferred to the electricity production process are raised. Therefore, it is uncertain whether the net effect of China-VI will be positive or negative, and it is necessary to carry out a precise assessment of it.

The object of this study is to bridge the above research gap by evaluating the emission reduction effects of the implementation of the latest vehicular emissions standard of CHINA-VI. To this end, we applied a difference-in-differences method to establish the policy measurement model on prefecture-level regulations with monthly panel data of 276 cities from 2018 to 2020. Four indicators related to air pollution were employed to measure the air quality of a city, namely, the AQI, PM_2.5_, PM_10_, and O_3._ To provide robust evidence, we also used a variety of estimation strategies such as PSM-DID and IV-2SLS to conduct a series of robustness tests. Moreover, we further investigated the heterogeneous environmental effects of CHINA-VI in different cities to identify the alternative or complementary effects between CHINA-IV and other related policies.

This study makes several contributions. First, the current body of evidence concerning the environmental impacts of regulations on vehicular emissions primarily originates from developed nations. However, due to differing contexts and institutional frameworks, the transferability of these findings to developing nations may be limited. Based on our current knowledge, this article represents the first attempt to examine the mitigating effects of CHINA-VI on air pollution at the prefecture level cities by using a quasi-natural experimental method, thus supplementing evidence from developing economies. Second, this article not only confirms findings similar to previous studies from developed countries that vehicular emissions standards have a mitigating effect on air pollution, but also provides some new insights for policy-makers to better understand the heterogeneous performance of CNINA-VI. For example, our results suggest that cities with lower levels of green finance development and public environmental concern showed a greater emissions reduction effect, indicating that the implementation of CNINA-VI can compensate for the negative impacts of insufficient green finance and public supervision on environmental performance in these cities. However, a greater emissions reduction effect was observed in smart cities than in non-smart cities, which means the digital and intelligent transformation of cities can effectively strengthen the effect of vehicular emissions regulation in addressing automobile pollution.

The remainder of this paper is structured as follows. The second section provides the policy background, the third section is the literature review and hypothesis, the fourth section presents the data sources and empirical strategies, the fifth section presents the results of the baseline regression and its robustness tests, the sixth section provides the heterogeneity in the performance of CHINA-IV among cities, and the seventh section provides a summary and recommendations based on the results of the study.

## Policy background

**Fig. 1:**
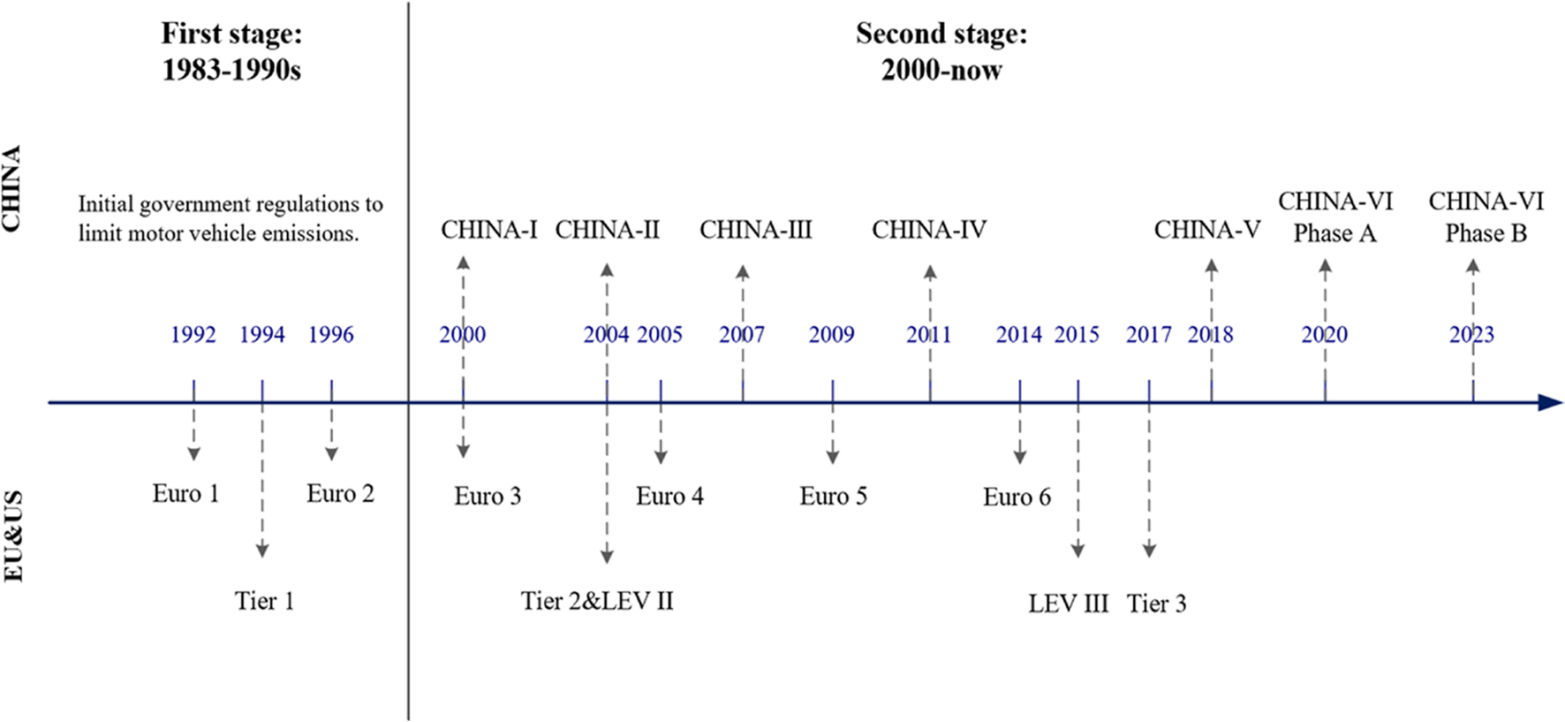
The development of vehicular emission standards in China, EU, and the USA

In terms of the development of vehicular emission standards, many countries have long been following the standards set by the EU and the USA, and China has also taken the EU vehicular emission standards as a reference for a long time. The EU standards are mainly realized by the emission regulations of the European Economic Commission (ECE) and the emission directives of the European Union. As shown in Fig. 1, the earliest European vehicular emissions standard—EU-I—was widely promoted in 1992, and the latest operating EU-VI standard was started in 2014, which has entered the fourth and final stage in 2020. In addition, the EU-VII standard has also been established and is expected to be implemented in 2025. In comparison, the full implementation of the EU-VI standard is 6 years earlier than the CHINA-VI standard; although the implementation time is later, the emission limits of CHINA-VI are lower than the Euro 6 based on reference from the latter. Besides, the end time of CHINA-VI also catches up with the pace of the EU-VI. In this phase, the gap between China’s emission standards and European emission standards will be smaller, and it is possible for China to exceed.

American vehicular emission standards are divided into two categories, the EPA standards of the Environmental Protection Agency and the CARB standards of the California Air Resources Commission. The EPA standards have undergone three phases, tier 1, tier 2, and tier 3. The tier 3 standards which are currently in force will be implemented in stages during 2017–2025. Some emission limits in tier 3 are much stricter than CHINA-VI, such as the required 0.02 g/mile (equivalent to 12.4 mg/km) of NOx emission, which is nearly 75% below the 50 mg/km limit in phase B of CHINA-VI. CARB’s vehicular emission standards, called LEV (low emission vehicles), are similar to EPA standards, but are more stringent than the latter. CA has implemented LEV III in stages since 2015 and plans to complete the whole process by 2025; what is more, LEV III is expected to be gradually unified with the EPA tier 3. In contrast to China’s vehicular emission standards, LEV III is also stricter than CHINA-VI, with the required limits close to zero emissions.

Compared with developed countries, China’s start time of establishing the systematic vehicular emission standards is late, and the entire process can be broadly categorized into two distinct stages: the first stage is 1983 to 1999, when China initially established vehicular emissions standard system; the second stage is 2000 to now, with stricter emission limits are introduced continuously and the emission quantity of all vehicle exhaust reduced observably.

In the first stage, China promulgated the first batch of automobile emission standards in 1983, which stipulated six standards, including the emission concentration of CO and HC of gasoline cars, and the free acceleration and full load smoke of diesel vehicles, which laid the foundation for China’s pollution control in the area of motor vehicular emissions. Following this, China released a set of regulations in the early 1990s to control pollutant emissions. The regulations’ main body included large gasoline vehicles, light vehicles, and motorbikes, and the specified restricted pollutants included CO, HC, and NOx. At this time, although these standards did not significantly tighten the emission limits, they requested production enterprises to improve product quality and technological level, in order to make motor vehicles reach higher production consistency when promoting emission reduction from the production technology level.

In the second stage, China entered a period of rapid growth in the automobile industry, and the accompanying air pollution problem was becoming more and more serious, so China began to implement stricter emission standards. Being complementary to China’s decision to reduce plumbum in gasoline, the Environmental Protection Department promulgated CHINA-I and CHINA-II in 1999, and these two initial emission standards referred to EU-I and EU-II; the move greatly promoted China’s motor vehicle pollutants emission control level. In 2005, to address the issue of air pollution stemming from vehicular emissions and foster sustainable growth in the automobile industry, the State Environmental Protection Administration of China introduced stricter standards—CHINA-III and CHINA-IV—which were implemented nationwide in 2007 and 2010, respectively. Eight years after the implementation of CHINA-IV, CHINA-V was put into effect. In addition to comprehensively lowering the previous emission limits, CHINA-IV also added the emission limits for non-methane hydrocarbon and PM particulate matter, which has fully exceeded the Euro-VI in pollutant emission limits. This latest standard includes phase A standard and phase B standard, which were implemented nationwide on July 1, 2020, and July 1, 2023, respectively. In fact, in order to accumulate experience, the Chinese government had already conducted pilot projects in 18 cities before launching phase A standards at the national level. For example, Beijing, Shanghai, Guangzhou, Shenzhen, and Tianjin have launched CHINA-IV 1 year ahead of the nationwide plan and directly skipped the phase A standard to implement the phase B standard.

## Literature review and hypothesis

### Literature review

Since Europe and the USA being the earliest regions in the world to implement vehicle emission standards, almost all of the existing practical and empirical knowledge in this field comes from research in these countries. It is convinced that light-duty diesel automobiles can lower nitrogen dioxide emissions to improve air quality under European and American emission requirements (Von Schneidemesser et al. 2017). Likewise, after evaluating the effectiveness of domestic emission standards for light-duty vehicles (LDVs) in reducing emissions in the USA, scholars found that when emission standards are upgraded from tier 1 to tier 2, the maximum daily 8-h ozone amount as well as the monthly average PM2.5 quantity in the eastern USA will decrease by 2022 (Vijayaraghavan et al. 2012). Furthermore, using global composition-climate models, scholars have predicted that the adoption of European vehicular emission standards in developing countries would result in substantial annual benefits, including reduced mortality from air pollution-related diseases, avoided food production reductions, and a slowing of the warming process (Shindell et al. 2011). Regarding how the emission reduction effect of vehicular emission standards interact, through a study in the European market, a discussion was derived on the ways that enterprises choose to comply with the new standards—which are primarily divided into changing prices, cutting back on fleet size, and using new technologies—determine the welfare impact of the emission standards (Reynaert 2014). It is further suggested that the new emission standards in the European market have the effect of encouraging businesses to innovate technologically. Similarly, other scholars found that the promotion of associated technological advancements and the restriction of gasoline fuels both contribute to the pollution reduction effect of US tier II emission standards (Parry et al. 2007).

In China, vehicular emissions were one of the main causes of increased air pollution in the late twentieth century (Hao et al. 2000). In order to reduce vehicle exhaust pollution, the Chinese government has implemented corresponding vehicular emission reduction measures since 1995 (Hao et al. 2006). Current research on China’s vehicular emission control policies has focused in part on analyzing the emission reduction impact through scenario simulations. Researchers have discovered that despite a huge increase in the number of motor vehicles, vehicular emission control methods still significantly reduce air pollution (Guo et al. 2013; Saikawa et al. 2011). By simulating the impact of major vehicular control policies implemented in China, researchers found that the implementation of CHINA-IV and CHINA-V is very important to reduce vehicular emissions, and the earlier the standards are issued, the greater the benefits will be (Huo et al. 2015; Jia 2022).

Much of the other literature on emission standards concentrated on the improvement of air quality due to the implementation, as well as the policies’ social and economic impacts. Between 2000 and 2005, the implementation of a series of motor vehicle emission standards resulted in some environmental benefits for China, with annual CO emissions decreasing from 586 to 324 kg per unit of vehicle and NOx emissions decreasing from 66.9 to 43.4 kg per vehicle, but the emission reductions from increased emission standards were offset to some extent by the increase in the number of motor vehicles (Jin et al. 2012; Wang et al. 2010). With the continuous upgrading of emission standards, average pollutant emissions from motor vehicles in China have shown a significant downward trend, with annual change rates of CO and VOC in the Beijing-Tianjin-Hebei (BTH) region from − 3.1 and − 4.4 to − 5.2% and − 6.9%, respectively, between 1999 and 2010 (Lang et al. 2012). Pollutant emissions from light vehicles decreased by 91–96% under the National IV standard, which was implemented in 2013, and its total emission reduction effect is very significant and has a notable beneficial impact on enhancing the urban air quality in China (Ji et al. 2011; Sun et al. 2017b). Aside from the positive environmental effects, upgrading and implementing motor vehicle emission standards has certain socioeconomic costs for China, such as disincentives to the automobile industry, higher labor costs, and a slight decrease in GDP (Liu et al. 2016). However, despite the high cost of emission control, it has been demonstrated that vehicle emission standards can achieve relatively significant emission reductions at a reasonable cost (Shao et al. 2001).

In terms of variability in the role of vehicular emission standards, scholars compared the Chinese provincial automobile exhaust comprehensive control policy effect and found that the emission standards are ineffective in controlling diesel vehicle emissions in plain areas, but only serve to reduce the amount of nitrogen oxide emissions (Zhang et al. 2019). In addition to emission standards, other vehicular emission reduction policies also have a positive effect on reducing air pollution (Zhang and Wang 2020), including setting oil prices (Xi and Liang 2015), driving restriction policies (Yi et al. 2018), and promoting new energy vehicles (Tan et al. 2018; Wang et al. 2021). Some scholars have pointed out that the implementation of such environmental regulations, while improving urban air quality, can also have the negative effects of traffic congestion and increased crime rates (Carrillo et al. 2018; Chu et al. 2019; Wang et al. 2019).

With regard to the current CHINA-VI, scholars point out that, although China’s motor vehicular emission standards have long lagged behind those of developed countries in Europe and the USA, the latest CHINA-VI has vigorously filled the gaps (Cai et al. 2017). The tailpipe emission restrictions of CHINA-VI are 50% more stringent than CHINA-V (Gong et al. 2017), and through the comparison with other countries, it is pointed out that this latest standard has introduced the light vehicle test cycle technology and the regulations to strengthen the evaporative emission control from the US standards; therefore, it is believed to be stricter than the European vehicular emission standards (Bao et al. 2017).

In summary, previous studies have proved that vehicular emission standards can significantly mitigate the pollutant emissions in vehicle exhaust or improve urban air quality. As yet, however, to our knowledge no research has been done into the emissions reduction effects of the newly implemented CHINA-VI. Also, studies on the heterogeneity of the effects of vehicular emission standards in China have mainly distinguished by physical and geographical factors, and less explored, and less focused on the alternative or complementary effects between CHINA-IV and other related policies. Therefore, this study attempts to bridge the above research gap by investigating how CHINA-VI affects Chinese prefecture-level cities’ ability to reduce atmospheric pollution. Furthermore, we also examined the heterogeneity in the performance of CHINA-IV among different types of cities from three aspects: the level of green finance development in cities, the level of public environmental concern in cities, and the construction of smart city, which can identify the alternative or complementary effects between different policies.

### Research hypothesis

Through the analysis of the previous literature review, urban air quality is anticipated to improve as a result of CHINA-VI; however, it is also a subject that has to be explored if the improvement effect will vary based on the city features. Therefore, this section follows with a theoretical analysis of the heterogeneity of CHINA-VI abatement effects.

#### The moderated role of green finance development

Green finance, which generally refers to green asset financing, credit, and investment that addresses environmental protection objectives, is an innovative financial approach that integrates revenue growth and environmental protection (Höhne et al. 2012; Muganyi et al. 2021; Soundarrajan and Vivek 2016). It is recognized that sustainable economic growth and environmentally friendly manufacturing are favorably impacted by the green finance policy (Cui et al. 2020; Hassan et al. 2022). China’s 13th Five-Year Plan advocates the establishment of a green finance system and encourages the private sector to take a more active role in sustainable development. Currently, thanks to a comprehensive green transformation financing model, China’s green finance is developing rapidly, making an important contribution to the structure upgrading and sustainable development of China’s manufacturing sector (Jin and Han 2018), and China has become one of the global leaders in green finance (Gallagher et al. 2018). Contrasted with conventional financial operations, green finance emphasizes ecological advantages and concentrates more on green industries. In the absence of intervention by green finance, financial institutions are more inclined to invest in traditional projects than in green projects, based on their preference for quick profits and their avoidance of potential risks in the face of emerging green technologies (Sachs et al. 2019; Yoshino et al. 2019). Returning to the impact of CHINA-VI on air pollution emissions, it can be surmised that regions with higher degrees of green financial development will be more inclined to receive financial support for green projects, so their original green project construction levels will be higher and the utilization of cutting-edge emission reduction technologies will be highly prevalent. For regions with lower levels of green financial development, their previous financial support for green projects is lower, the application of green emission reduction technologies is less, and pollution levels tend to be more severe, while the mandatory upgrade of vehicle emission standards forces them to cut pollution emissions to the same level as regions with high green financial development; these regions will then have a greater abatement effect.

#### The moderated role of public environmental concern

Public environmental concern (PEC) generally refers to the level of public awareness of environmental issues and willingness to take action to address them (Dunlap and Jones 2002). Previous studies have demonstrated the beneficial influence of PEC on environmental protection behavior, and there is currently considerable attention on the significant contribution PEC makes to enhancing eco-efficiency and fostering sustainable social development (Minton and Rose 1997; Sun et al. 2017a). PEC can strengthen the regulatory channel for pollution control enforcement by collective protest to reveal the effects of pollution. Such attention and protest from the public will increase the social exposure of an enterprise’s environmental pollution behavior, expand its social influence, lessen its influence in negotiations with regional environmental authorities, and thereby provide an opportunity for regulators to increase pollution penalties (Wang et al. 2003; Wang and Wheeler 2005). Thus, PEC drives up the additional cost of pollution, increasing pressure to cut emissions (Liu and Mu 2016). Based on companies’ concerns about increasing pollution costs and corruption of their social influence, it can be speculated that in regions with higher PEC, producers will strive harder to enhance the sustainability of their production processes, such as adopting cleaner production technologies, improving energy utilization efficiency, and investing in green projects. Whereas, in regions with lower PEC, their initial pollution situation will be more serious due to the insufficient level of public monitoring. With the uniform implementation of CHINA-VI, high PEC areas and low PEC areas face the same mandatory emission limits, and low PEC areas certainly need to make greater emission reductions to meet the national standards.

#### The moderated role of smart city construction

The attention to sustainability in the urban development process has gradually influenced the city planning (Goonetilleke et al. 2014; Zhao 2011), yet smart cities are an important solution to guarantee sustainable development (Toli and Murtagh 2020; Wang et al. 2022). Smart cities are considered to be technologically advanced, environmentally friendly, and efficient (Vanolo 2014), which can focus on the problematic frontiers of urban ecological conservation, economic development, and social management to provide efficient solutions (Yigitcanlar 2016). Benefiting from features such as smart government, smart economy, and smart environment (Caragliu et al. 2011; Lombardi et al. 2012), smart cities will have better resource allocation mechanisms, higher levels of innovation, and stricter environmental targets, which will increase efficiency and sustainability in administration and production (Lazaroiu and Roscia 2012; Paroutis et al. 2014; Qian et al. 2021). It can be presumed that regions where smart cities are being built have a higher level of internal urban management and resource coordination, as well as a greater capacity for green innovation and easier access to support for green projects. Due to this comprehensive urban system building, smart cities are also more likely to have positive additive effects through internal inter-system coordination when environmental regulation occurs; in other words, the abatement effect of CHINA-VI will be more significant in the smart cities.

The following hypotheses are put forth in this paper based on the analyses previously mentioned.

- Hypothesis 1: CHINA-VI can produce abatement effects in Chinese prefecture-level cities.
- Hypothesis 2: In the context of the implementation of CHINA-VI, cities with lower levels of green finance will have a greater emission reduction effect.
- Hypothesis 3: Cities with lower levels of public environmental concern will produce greater emissions reductions under the implementation of CHINA-VI.
- Hypothesis 4: The abatement effect generated by CHINA-VI will be more significant in cities that participate in the construction of smart cities.

## Method and data

### Identification strategy and model setting

The empirical strategies of the present study are carried out in three steps. First, the DID method was employed to identify the average treatment effects of CHINA-IV on cities’ air quality. Second, the propensity score matching (PSM) method was integrated with DID regression to address potential sample self-selection bias, as well as a series of placebo tests were conducted to eliminate interference from other policies. Third, the heterogeneity of the treatment effects of CHINA-IV in different types of cities was examined in the last step.

The basic principle of the DID method is to treat exogenous policy shocks as a quasi-natural experiment, so that researchers can observe the changes in outcomes of individuals receiving policy intervention before and after policy intervention (Wen et al. 2021; Yang et al. 2021), namely, the average treatment effects on the treatment group (ATT). Compare to the ordinary least squares estimation method, an attractive feature of DID estimation is that it can remove the effects of unobserved confounders arising from time-invariant differences between the comparison groups, which greatly mitigate the problem of endogeneity (Zhang et al. 2022). As introduced in the policy background, 18 cities launched the CHINA-VI standard in 2019 (1 year ahead of the national plan), while other cities launched the CHINA-VI standard in 2020. This provides a quasi-natural experiment for us to use the DID method to identify the average treatment effects of CHINA-VI.

Specifically, due to the severe lack of data on air quality and pollution emissions in some cities, we ultimately selected 276 cities as the research samples. As shown in Fig. 5 in the Appendix, 18 cities that initiated the CHINA-VI standard 1 year ahead of the national plan (i.e., 2019) were considered the treatment group, while the remaining 258 cities were considered the control group. Thus, the average treatment effects of CHINA-VI can be expressed as

1 $$AQI=\alpha +\gamma ESV{I}_{\text{it}}+\beta {X}_{it}+{\mu }_{i}+{\eta }_{t}+{\varepsilon }_{it}$$

where the explanatory variable AQI (air quality index) was released as a new air quality evaluation standard by China’s government in 2012, which is regarded as a comprehensive indicator that reflects the level of air pollution (Xue et al. 2019). The AQI data selected for this study were all obtained from the China Air Quality Online Monitoring and Analysis Platform. *ESVIit* is a dummy variable representing the status of the policy implementation, i.e.,

2 $$ESV{I}_{it}=treate{d}_{i}\times perio{d}_{t}$$

If the observation city implements the CHINA-IV standard in 2019, *treated*_*i*_ will take the value of 1, and 0 otherwise; *period*_*t*_ equals 1 after the policy start point, i.e., July 2019, and 0 at otherwise. *X*_*it*_ is a series of covariates for urban characteristics, *μ*_*i*_ is individual fixed effect for the city, *η*_*t*_ is time fixed effect, and *ε*_*it*_ is an error term.

However, in the DID setup, we need to assume that without the policy intervention, outcomes for the treatment and control groups would have followed parallel trajectories over time (Kreif et al. 2016). Whether the parallel trend assumption holds in applied settings is often a major uncertainty (Kreif et al. 2016), which means that we need to conduct the parallel trends testing when using the DID method to ensure its applicability in this article.

Moreover, even if the parallel trend assumption is valid, another potential challenge that cannot be ignored for DID estimations is that individuals participate in the CHINA-IV standard may be the result of their own choice. For example, cities with stricter environmental regulations may initiate CHINA-IV earlier than other cities. Under this situation, the difference obtained by directly subtracting the outcome of the treatment group from the outcome of the control group does not represent the net treatment effect, as the average treatment effect (ATE) is composed of two parts: ATT and selection bias. In the following empirical analysis, we adopted two approaches to address this issue. On the one hand, we tested whether the treatment group receiving policy intervention was a random process through a placebo test. On the other hand, we first used the PSM method to search for individuals with similar conditions, abilities, and backgrounds before policy intervention. The only difference is that some individuals have implemented CHIAN-IV, while others have not. On this basis, the DID method introduced above was used to compare the differences of air quality between these two groups.

### Covariates and other variables

This paper selected the following covariates at the prefecture city level for reference: (1) The level of economic development (lnrpgdp), measured using real GDP per capita in logarithmic form; (2) urban greening level (lngreenl), measured using the city’s greening coverage of built-up areas; (3) the level of government intervention (fiscal), the calculation involved using the proportion of local government budget revenue to expenditures (Gao et al. 2022); (4) public transportation level (bus), measured by the total annual public bus and tram passenger traffic; (5) science and technology expenditure (sciexp), the measurement was based on the amount of expenditure on science and technology within the general public budget expenditure of local governments; (6) the deposit status (deposit), measured by the year-end deposits in RMB held by financial institutions; (7) temperatures (AT), measured by average monthly temperature; (8) wind speeds (AWS), measured by average monthly wind speeds; (9) precipitation (AR), measured by the average monthly amount of precipitation; (10) air pressure (AP), measured by average monthly air pressure. Table 1 presents the descriptive statistics, which include observations, means, standard deviations, and minimum as well as maximum values, of the key variables utilized in this paper.

**Table 1:** Description statistics of the variables

	(1)	(2)	(3)	(4)	(5)
Variables	*N*	Mean	sd	min	max
AQI	8556	64.2380	27.2595	14.4582	207.8546
did	8556	0.0273	0.1631	0.0000	1.0000
lnrpgdp	8521	10.8802	0.5101	9.4056	12.1853
lngreenl	8208	3.7125	0.1733	− 0.9416	4.5413
fiscal	8544	0.4086	0.2090	0.0571	1.0857
bus	8229	19.4549	36.4415	0.1610	318.9750
sciexp	8544	177.5696	525.7781	1.2310	5549.8170
deposit	8532	6.3398	14.7270	0.3466	181.1056
AT	8556	14.7696	10.5134	− 24.9813	30.7434
AWS	8556	2.2513	0.5617	0.9182	5.1278
AR	8556	86.3115	82.6319	0.0000	663.6222
AP	8556	966.9919	57.3525	753.5705	1030.0440

### Data sources

From 2018 to 2020, a total of 276 cities in China were selected for this paper. The statistical variables’ raw data was procured from various sources such as the China Statistical Yearbook, China City Statistical Yearbook, and China Environmental Statistical Yearbook, in addition to provincial and municipal statistical yearbooks. Any missing values were interpolated to ensure completeness.

## Empirical results analysis

### The AQI inhibiting effect of the CHINA-VI

Table 2 provides the pollutant reduction effects under the CHINA-VI; the conclusion of this baseline regression is drawn with the DID method. According to column (1), when no covariates are factored in, the estimation coefficient of ESVIit shows a significant negative correlation at the 1% level. This finding supports the link between implementing CHINA-VI and the decrease of AQI in China. Covariates are introduced in columns (2), (3), and (4) based on the setting of column (1), the estimation coefficients fail to remain statistically significant when only time fixed effects are added; however, the results in columns (2) and (3) still show the emission reduction effect of CHINA-VI at the 1% significant level. Column (5) simultaneously incorporates time and city fixed effects, as well as covariates, thereby increasing the credibility of the regression results. In column (5), the estimated result reaches a statistical significance level of 1%, demonstrating that the adoption of the new CHINA-VI standard can cut AQI by 4.74, indicating a considerable decrease in air pollution levels. These findings align with prior research and support Hypothesis 1.

**Table 2:** Baseline regression results (AQI)

	(1)	(2)	(3)	(4)	(5)
*ESVIit*	− 6.0311***	− 5.2735***	− 5.2736***	0.9595	− 4.7405***
	(1.4735)	(1.6247)	(1.9635)	(3.0293)	(1.7775)
lnrpgdp		− 15.1417***	− 16.7579***	− 9.2443***	− 7.1383***
		(1.6794)	(2.2787)	(3.0791)	(1.9568)
lngreenl		− 0.5929	− 0.8238	3.461	− 1.1747**
		(0.9210)	(1.1862)	(3.2523)	(0.4649)
bus		0.0424*	0.0339	− 0.0086	− 0.0188
		(0.0258)	(0.0402)	(0.0390)	(0.0383)
fiscal		49.8662***	53.8085***	36.0517***	5.3759
		(4.7940)	(7.8989)	(8.0538)	(7.5473)
deposit		− 0.1565	− 0.2495	0.0948	0.1307
		(0.0963)	(0.2510)	(0.0701)	(0.1549)
sciexp		0.0004	0.0016	− 0.0049**	0.0031***
		(0.0016)	(0.0020)	(0.0020)	(0.0012)
AT		− 1.4002***	− 1.4159***	− 1.1763***	− 1.4462***
		(0.0683)	(0.1215)	(0.0862)	(0.1239)
AR		− 0.0481***	− 0.0453***	− 0.0853***	− 0.0442***
		(0.0033)	(0.0044)	(0.0064)	(0.0044)
AP		0.0504***	0.0557	0.0559***	0.0140
		(0.0170)	(0.1466)	(0.0155)	(0.1478)
AWS		− 0.7030	0.0586	− 5.2844***	0.2743
		(0.7876)	(1.0496)	(1.4348)	(1.0327)
_cons	64.4029***	189.0895***	199.8741	120.7582***	154.7602
	(0.0403)	(22.2644)	(147.2394)	(33.4372)	(146.5585)
Time fixed effects	Yes	No	No	Yes	Yes
City fixed effects	Yes	No	Yes	No	Yes
Observations	8556	7934	7934	7934	7934
R-squared	0.3990	0.5254	0.7084	0.4261	0.7147

Standard errors are in parentheses, *** *p* < 0.01, ** *p* < 0.05, * *p* < 0.1

### Parallel trend test

The explanatory variables must meet the parallel trend assumption for both the treatment and control groups in order to analyze policy impacts using the DID method; it implies that if a policy shock did not occur, both groups’ dependent variable trajectories should have been the same.

In this paper, the previous period of the policy implementation is considered the base period. We create cross-multiplication terms for a 10-month interval, linking the time dummy variables and the policy dummy variables, spanning from 4 months before implementation to 6 months following implementation (Beck et al. 2010; McGavock 2021). The model is specifically defined as follows:

3 $$AQI=\alpha +\sum_{s=2}^{4}{\beta }_{pre\_s}{D}_{pre\_s}+{\beta }_{current}{D}_{current}+\sum_{s=1}^{6}{\beta }_{post\_s}{D}_{post\_s}+\varphi Contro{l}_{it}+{\mu }_{i}+{\eta }_{t}+{\varepsilon }_{it}$$

**Fig. 2:**
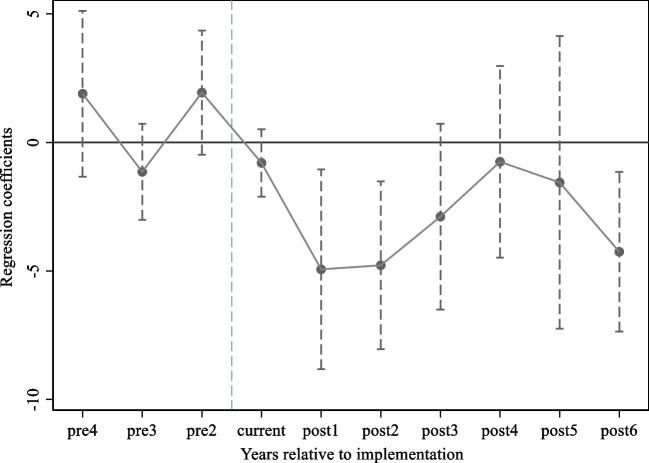
Estimated coefficients before and following the implementation time

${D}_{pre\_s},{D}_{current},{D}_{post\_s}$ Denotes the cross-multiplicative term for the time dummy variable and the policy dummy variable for the pre-, current-, and post-implementation periods of CHINA-VI. The relevant coefficients are ${\beta }_{pre\_s},{\beta }_{current},{\beta }_{post\_s}$ correspondingly; Fig. 2 exhibits the outcomes. Prior to the introduction of CHINA-VI, the time-varying trend of AQI did not appear statistically significant, and the coefficients are approximately zero. Further, the dynamic test’s subsequent regressions demonstrate that starting from the month following the adoption of the new standards, the CHINA-VI diminished the AQI of the pilot cities significantly. The results satisfy the parallel trend hypothesis.

### Robustness tests

#### Placebo test

**Fig. 3:**
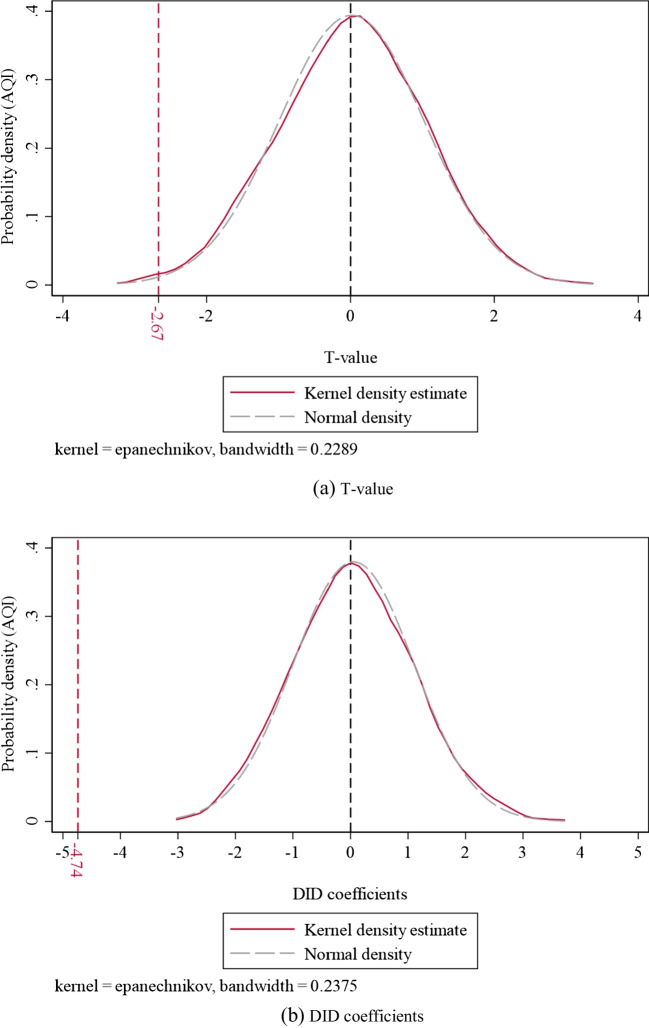
The *t* value and coefficients empirical cumulative distribution of placebo test

The placebo test demonstrates the accuracy of policy impact evaluations by excluding the spurious regression brought on by missing variables. Across all pilot cities and time frames discussed in this article, unduplicated random sampling is carried out (Cao et al. 2021). A random set of 18 cities was chosen each time as the virtual experimental group, while the remaining 258 cities were used as the virtual control group. Additionally, a random selection was also made for the period when the policy was put into effect. This procedure is carried out 1000 times to obtain 1000 virtual DID regression estimates. Figure 3 displays the test outcomes, featuring the red solid line that portrays the probability density distribution of *t*-value and DID coefficients during the placebo test, while the dashed line represents the normal density distribution. The left red vertical dashed line indicates the baseline regression’s *t*-value and DID coefficients, which are situated in the low tail of the probability density distribution in the placebo test. The means of the results obtained from the placebo test are far from the true values; this indicates that the policy effects are not influenced by other unobserved factors. The baseline regression findings in this study therefore pass the placebo test.

#### PSM-DID

**Fig. 4:**
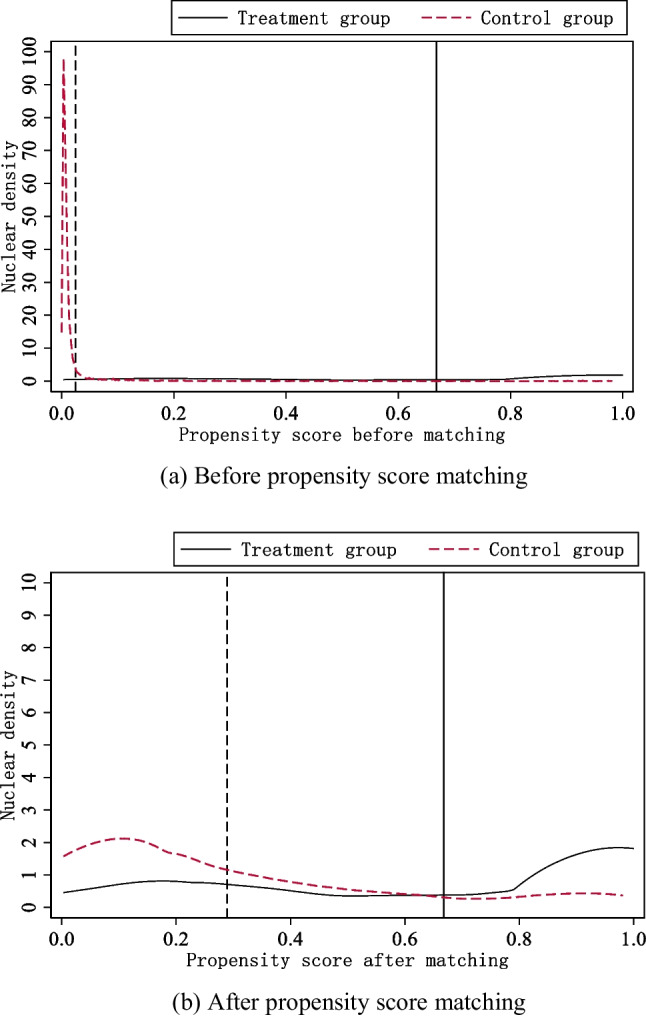
Kernel density before and after propensity score matching

**Fig. 5:**
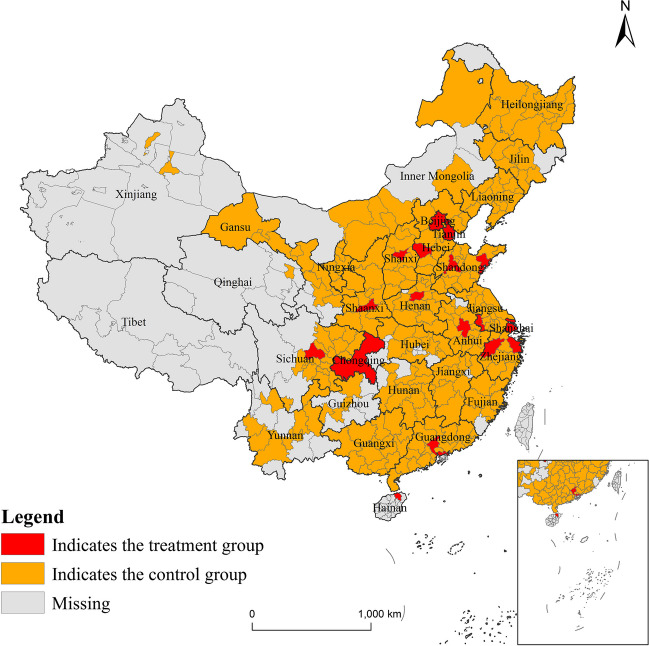
The distribution of the treatment and control groups

To eliminate the sample self-selection bias, cross-sectional propensity score matching (PSM) was initially conducted for the entire sample using the *k*-nearest neighbor method. This implies that the policy effects measured after using the PSM method are possible to exclude the interference of unobservable factors, making the results more robust. By comparing the estimates of the propensity scores before and after matching, it can be shown in Fig. 4 that the kernel density curves’ average distance between the treatment and control groups exhibited a decrease. This suggests that the matching was successful. After conducting separate DID regressions utilizing samples with non-null weights, samples that satisfied the common support hypothesis, and frequency weighting method, the results shown in Table 3 remained robust even after accounting for selection bias. Furthermore, it was found that CHINA-VI significantly contributed to suppressing air pollutant emissions, as evidenced by a decrease of AQI by 6.42, 5.66, and 7.74, respectively.

**Table 3:** PSM-DID regression results (AQI)

	(1)	(2)	(3)
	Samples with non-null weights	Samples satisfying the common support hypothesis	Frequency-weighted regression
*ESVIit*	− 6.4172**	− 5.6592**	− 7.7438**
	(− 2.3607)	(− 2.1869)	(− 2.6380)
Covariates	Yes	Yes	Yes
Year fixed effects	Yes	Yes	Yes
City fixed effects	Yes	Yes	Yes
Observations	602	2008	836
R-squared	0.7711	0.7574	0.7506

Standard errors in parentheses; ***p < 0.01, **p < 0.05, *p < 0.1

#### IV-2SLS

To exclude the influence of endogenous problems, this article also conducts the instrumental variable (IV) method (Cai et al. 2016; Liu et al. 2023; Yang et al. 2022). The IV (*historypost*) is obtained by cross-multiplying the ancient capital variable with the policy time dummy variable *period*_t_, and the two-stage least squares (2SLS) is used to conduct the regression. The search for instrumental variables from the perspective of historical data is a common practice in the existing literature, since existing historical facts do not have an impact on current environmental data (Acemoglu et al. 2001; Chen et al. 2020). It means that using historical data as instrument variables can effectively avoid endogeneity due to reverse causality of the dependent variable with the independent variable. The geographic location of ancient capitals is highly correlated with the economic and political circumstances of the entire nation at the time (Zhao and Hu 2019). As the political, economic, and cultural centers of the ancient Chinese dynasties, the cities that had been chosen as ancient capitals have a more profound political and cultural heritage. Being the basis of political and cultural development, the local economic development is also generally relatively leading, with a more developed industrial level, and more likely to take the initiative to implement CHINA-VI in advance to play an exemplary role, this meets the correlation requirement of the instrumental variable. Since the general environmental pollution in ancient times would not be left to modern times, this IV satisfies the exogeneity condition with the explanatory variables. According to Table 4, both of the regression results remain statistically significant, and the coefficient corresponds with the baseline regression, which verifies the reliability of the result of the baseline regression.

**Table 4:** 2SLS regression result of instrumental variable method (AQI)

	(1)	(2)
	Phase I	Phase II
historypost	0.1993**	
	(0.0856)	
*ESVIit*		− 28.3300***
		(6.4588)
Covariates	Yes	Yes
Year fixed effects	Yes	Yes
City fixed effects	Yes	Yes
Observations	7934	7934
*R*-squared	0.1392	0.0144

Standard errors in parentheses; ***p < 0.01, **p < 0.05, *p < 0.1

#### Substitution of dependent variable

The AQI values are calculated from the concentrations of several air pollutants. To put the validity of the initial regression results to the test further, three pollutant concentration values of PM2.5, PM10, and O_3_, which constitute the AQI sub-indicators, are selected as substitute variables in this paper to replace the original explanatory variable AQI in the baseline regression. As evidenced by the findings in Table 5, the three pollutant concentrations produced significant reductions under the impact of the CHINA-VI, and the results remain statistically significant, which further confirms the reliability of the baseline regression result.

**Table 5:** Regression results of substitution variables

	(1)	(2)	(3)
	o3_month	pm10_month	pm25_month
*ESVIit*	− 2.9937*	− 6.3990**	− 3.6046***
	(1.6089)	(2.9010)	(1.3782)
Covariates	Yes	Yes	Yes
Year fixed effects	Yes	Yes	Yes
City fixed effects	Yes	Yes	Yes
Observations	7934	7934	7934
*R*-squared	0.7077	0.7165	0.7071

Standard errors in parentheses; ***p < 0.01, **p < 0.05, *p < 0.1

## Heterogeneity analysis

By tightening the emission limits for motor vehicles, CHINA-VI has a dampening effect on vehicles’ pollutant emissions, thereby reducing the concentration of air pollutants throughout the city. Although the pollutant reduction effect of this new standard is clear, the heterogeneity that exists among different types of cities will produce variations in the reduction effect of this standard. To understand these differences, we further conducted the following heterogeneity analysis.

1. The impact of green finance development. Drawing on previous studies (Liu and He 2021), we construct a green finance development index for Chinese prefecture-level cities by the entropy method during 2018–2020. Then, we classified areas with a green finance index greater than the average as high green finance cities, conversely, as low green finance cities. As seen in columns (1) and (2) of Table 6, both of the regression coefficients remain negative, which is consistent with benchmark regression. The coefficients for cities with high green finance levels are not significant, in contrast, the result is statistically significant at the 1% level in low green finance cities, which also has a larger absolute value of coefficient; this suggests that CHINA-VI will produce greater emission reductions in cities with low green finance level, consisting with Hypothesis 2. Compared to high green finance cities, cities with low green finance have been underinvested in green projects, and their emission reduction technologies are relatively backward and more polluting, so they need to carry out stricter emission reduction measures to meet the emission limits of CHINA-VI. From this perspective, CHINA-VI accelerates the acceleration of motor vehicle emissions to a uniform and more stringent standard in different cities, compensating for the high emissions caused by insufficient investment in green projects in some regions, and better contributing to China’s sustainable development.
2. The impact of public environmental concern. The search frequency of environment-related keywords can reflect the level of public concern about environmental issues in a local area (Li et al. 2021), and as the most widely used search engine in China, the Baidu index provided by Baidu can provide a more realistic picture of the local PEC situation (Wu et al. 2021). In reference to other research (Liu et al. 2023; Yu and Jin 2022), the Baidu indices of 25 environment-related terms are summed to obtain the proxy indicator for PEC in this section. By using the total monthly mean values of all samples in 2018 as the basis for classification, we categorize areas above the mean value as high PEC cities and vice versa as low PEC cities. The results in columns (3) and (4) of Table 6 shows that the abatement effect of CHINA-VI is significantly stronger in low-PEC cities than in high-PEC cities. The regression coefficient in the third column is not only statistically significant at 5% level, but its absolute value is also larger than that in high PEC cities, as opposed to high PEC cities, where the regression results are not significant, although they are in the same direction as the main regression. Consistent with Hypothesis 3, it is clear from the regression results that the social impact and regulatory channels of emissions behavior are narrower and the cost of pollution is lower in low PEC regions due to the lack of prior public attention. In the absence of external intervention, local firms are less likely to invest capital in green technology upgrades and green project construction that have lower returns and require longer time spans, resulting in more serious pollution overall. When CHINA-VI began to be implemented, it put mandatory pressure on low PEC areas to reduce emissions, forcing them to meet the same emission limits as high PEC areas, making the actual reduction in low PEC areas more significant.
3. The impact of smart city construction. China began to carry out the pilot construction of smart cities in 2012, and by 2014, a total of 94 cities had in the pilot list. In this section, the 276 samples are divided into two groups by the construction status of the smart cities pilot, and then the baseline regression is conducted separately. Through columns (5) and (6) in Table 6, the results all show negative values, consistent with the direction of the baseline regression. However, the result is much more significant when cities participate in smart city pilot policies, which demonstrate that there is a greater abatement effect in the smart cities under the implementation of CHINA-VI; thus, hypothesis 4 is confirmed. Technological progress, information disclosure, and coordinated resource allocation are important tools to resolve the contradiction between environment and growth in economic development, and smart city construction provides the stage for that, and its complementary role to pollution emission policies is important in the current society that emphasizes both digitalization and sustainable development.

**Table 6:** Heterogeneity results of green finance development

	(1)	(2)	(3)	(4)	(5)	(6)
	Low green finance cities	High green finance cities	Low PEC cities	High PEC cities	Smart cities	Non-smart cities
*ESVIit*	− 8.7267*** (3.1569)	− 3.4651 (2.2217)	− 5.4682** (2.6511)	− 2.4055 (2.1546)	− 6.1329** (2.7516)	− 3.1138 (2.3112)
Covariates	Yes	Yes	Yes	Yes	Yes	Yes
Year fixed effects	Yes	Yes	Yes	Yes	Yes	Yes
City fixed effects	Yes	Yes	Yes	Yes	Yes	Yes
Observations	2960	4974	5485	2449	2914	5020
*R*-squared	0.6944	0.7287	0.7318	0.6766	0.6879	0.7292

Standard errors in parentheses; ***p < 0.01, **p < 0.05, *p < 0.1

## Conclusions and recommendations

Vehicle exhaust has grown to be a significant contributor to ambient air pollution in China as a result of the rapid expansion of the demand for private motor vehicles. Using monthly panel data from 276 cities from 2018 to 2020, this study is the first to investigate the vehicular emission reduction effects of the latest CHINA-VI standard by employing the DID method. The results show that the AQI has significantly decreased by 4.74, and the concentration of PM_2.5_, PM_10_, and O_3_ has decreased by 3.6 μg∕m^3^, 6.4 μg∕m^3^, and 3.0 μg∕m^3^, respectively, after the implementation of CHINA-VI. The results are robust to several robustness checks such as placebo testing and controlling for the sample self-selection bias. Previous studies in this field mainly focused on the performance of the EU standards and the US standards (e.g., Von Schneidemesser et al. 2017; Vijayaraghavan et al. 2012; Shindell et al. 2011; Reynaert 2014). Although our findings are similar to these studies, we supplemented evidence from developing economies to support that stricter vehicular emission standards can lead to better environmental performance.

Besides, the further heterogeneity analysis suggests that cities with lower levels of green finance development and public environmental concern showed a greater emissions reduction effect, while cities located in the pilots of smart city creation showed a greater emission reduction effect than non-smart cities. Compared to studies examining the environmental performance of China’s early vehicular emission standards (e.g., Jin et al. 2012; Wang et al. 2010; Lang et al. 2012; Ji et al. 2011; Sun et al. 2017b), we provided some new insights for policy-makers to better understand the substitution effects or complementary effects between CHINA-IV and other related policies regarding the role in improving air quality. Specifically, on the one hand, there is a certain degree of substitution effect between the implementation of the CHINA-VI standard and policies aiming at strengthening green finance development and public environmental concern. This indicates policy-makers can compensate for the negative impacts of insufficient green finance and public supervision on environmental performance by expanding the coverage of the CHINA-IV standard. On the other hand, there is a certain degree of complementary effects between the implementation of the CHINA-VI standard and polices supporting the construction of smart cities. This indicates to policy-makers that accelerating the digital and intelligent transformation of cities can effectively strengthen the positive role of CHINA-IV in addressing automobile pollution.

From the perspective of future research, this study has several limitations. It will be interesting and necessary for future research to expand our findings from two aspects. First, what are the underlying mechanisms of the CHINA-IV standard in improving cities’ air quality? In theory, on the production side, it may force automakers to enhance green innovation aimed at controlling exhaust emissions; on the consumer side, it may also mitigate consumers’ demand for gasoline vehicles or increase the probability of taking public transportation for travel. Second, what is the relationship between the CHINA-IV standard and the promotion policies for new energy vehicles? In other words, the increasing substitution of new energy vehicles for gasoline vehicles may offset the environmental performance of the implementation of CHINA-IV, given that that China’s electricity structure has not completely shaken off its dependence on thermal power.

## Data Availability

The data used in this paper can be obtained from the corresponding sources provided in the paper.

## Appendix

Figure 5
